# RETRACTED ARTICLE: Efficacy of skeletally anchored modified leaf expander versus modified conventional Hyrax in maxillary molar distalization: a randomized clinical trial

**DOI:** 10.1186/s12903-026-07836-3

**Published:** 2026-03-11

**Authors:** Mohamed Elsaharty, Mahmoud M. Fathy Abo-Elmahasen, Nouran M. Eissa, Mohamed Elbialy

**Affiliations:** 1 Department of Orthodontics, Faculty of Dentistry, Tanta University, Tanta, Gharbia Governorate Egypt; 2 Department of Orthodontics, Faculty of Dental Medicine (Boys, Cairo), Al-Azhar University, Cairo, Egypt; 3 Department of Orthodontics, Faculty of Dentistry, Mansoura University, Mansoura, Egypt

The editors have retracted this article. After publication, concerns were raised that this article uses an inaccurate clinical trial registration number and that the patient shown in Figure 5 belongs to a different study. The content of the article has been removed to respect the privacy of the patient and the rights of the image holder. An investigation by the publisher also found that the ethical approval claimed in the paper is not appropriate. The authors were unable to provide that they had prior ethical approval for this research from Tanta University. Additionally, Nouran Eissa states that she was not shown the final draft of the manuscript before it was submitted to the journal.

Nouran Eissa agrees to this retraction. Mohamed Elsaharty, Mahmoud M. Fathy Abo-Elmahasen, and Mohamed Elbialy disagree with this retraction.

